# Compact FPI-Based Fiber Optic Humidity Sensors Functionalized with PMMA/PVA/PEG

**DOI:** 10.3390/polym17212810

**Published:** 2025-10-22

**Authors:** Hongtao Dang, Fujing Chen, Jin Li, Fuhua Liu, Jianye Yang

**Affiliations:** 1 Shaanxi Engineering Research Center of Controllable Neutron Sources, School of Electronic Information, Xijing University, Xi’an 710123, China; 20170117@xijing.edu.cn (H.D.); 2308540606011@stu.xijing.edu.cn (F.C.); liufuhua@xijing.edu.cn (F.L.); 20200062@xijing.edu.cn (J.Y.); 2 College of Information Science and Engineering, Northeastern University, Shenyang 110819, China

**Keywords:** optical fiber sensors, humidity sensors, polymer materials, microfiber integration

## Abstract

The Fabry–Pérot interferometer (FPI) structure has been designed and fabricated through the heterogeneous splicing of single-mode fiber to hollow-core fiber, coupled with precision length cutting. Humidity sensitive materials of polymethyl methacrylate (PMMA), polyvinyl alcohol (PVA), and polyethylene glycol (PEG) film have been elaborated via a dip-coating and withdrawal technique, enabling the development of three distinct FPI-based fiber optic humidity sensors. Experimental data revealed that the PMMA-coated FPI sensor demonstrated the lowest sensitivity to humidity variations, while the PEG-functionalized FPI exhibited a sensitivity approximately an order of magnitude higher than that of PMMA. The proposed fiber optic humidity probe features a compact design, simplified fabrication workflow, and robust compatibility with spatially restricted, integration-demanding, or electrically hostile environments unsuitable for conventional sensor deployment.

## 1. Introduction

Fiber optic sensors have garnered extensive attention across diverse industries due to their compact size, lightweight, flexible configurability, high portability, low energy consumption, immunity to electromagnetic interference, corrosion resistance, intrinsic safety, and capacity for long-distance signal transmission [1]. These attributes render them indispensable in specialized domains, including aerospace, petrochemicals, nuclear engineering, and biomedical applications [2,3]. As an advanced sensing paradigm, fiber optic sensing technology utilizes optical fibers as primary information transmission media and light waves as information carriers. It operates by converting variations in environmental parameters (e.g., refractive index, electric current, temperature, humidity, concentration, and stress) into detectable alterations of optical signal characteristics (including intensity, polarization state, wavelength, and amplitude) within sensing structures [4]. Through appropriate demodulation techniques, these optical signal modulations can be quantitatively analyzed to achieve high-sensitivity measurements of physical and bio-chemical parameters [5].

High-performance fiber optic humidity sensors demonstrate substantial application potential in environmental monitoring [6], food safety assurance [7], and food processing [8]. Research priorities primarily focus on three critical aspects: sensitivity enhancement, measurement range expansion, and temperature cross-talk mitigation [9]. Current investigations address these challenges through three principal approaches: (1) Specialized optical fiber architectures: Implementation of polymer optical fibers (POF) [10], photonic crystal fibers (PCF) [11], and tilted fiber Bragg gratings (TFBG) [12], which exhibit unique optical properties for humidity detection. (2) Fiber structure modification: Application of post-processing techniques such as tapering, polishing, etching, and fusion splicing to optimize light-matter interactions and surface characteristics [13]. (3) Humidity sensitive material integration: Given the inherent hygroscopic limitations of silica (the primary constituent of optical fibers), surface or end-face functionalization with polymer materials represents a crucial strategy for performance improvement [14]. This integration enhances moisture adsorption capabilities and facilitates efficient optical parameter modulation in response to humidity variations.

Organic polymeric materials modulate their volumetric dimensions and refractive indices through ambient moisture absorption [15], operating via two distinct adsorption mechanisms: physical adsorption is governed by van der Waals interactions between polymer matrices and water molecules [16], enabling rapid moisture diffusion with superior reversibility and repeatability, as exemplified by agarose; chemical adsorption involves hydrogen bond formation between hydrophilic functional groups (e.g., carboxyl and amino groups [17]) and polar water molecules, typified by polyvinyl alcohol (PVA) [18]. Crucially, optimal polymer materials must exhibit stable physicochemical properties while achieving reversible volumetric and refractive index modulation through moisture adsorption–desorption cycles [19], thereby enhancing sensor performance without compromising other operational parameters. By elaborating PVA film onto the side-polished polymer fiber, Wang et al. demonstrated a humidity sensor with an average sensitivity of 4.98 nm/% RH [20]. The humidity sensitivity for one micro-bend fiber structure has also been improved to −0.6 nm/%RH after coating by PVA film [21]. Polymethyl methacrylate (PMMA) porous film functioned fiber Bragg grating [22] and polyethylene glycol (PEG) coated Fabry–Pérot fiber tip [23] have been proposed to measure the environmental humidity.

This work compared the humidity sensing performance of three different polymer materials, named PMMA, PVA, and PEG, using a Fabry–Pérot interferometric (FPI) based on cascaded-spliced fiber structure. To develop an FPI and generate the optical interference phenomena, a fiber optic sensing system requires two mirrors, which means that there should be a significant difference in refractive index inside and outside the Fabry–Pérot (FP) cavity. Therefore, the FP cavity of fiber optic sensors is usually constructed from micropores, microbubbles, or thin films made of materials different from the optical fiber. Compared with the traditional single mode fiber (SMF) and multi-mode fiber (MMF), hollow core fiber (HCF) has a significant difference in refractive index compared to the optical fiber. Meanwhile, the length of the HCF can be clearly observed under a microscope and finely cut into the required length [24,25].

## 2. Materials and Methods

**Figure 1:**
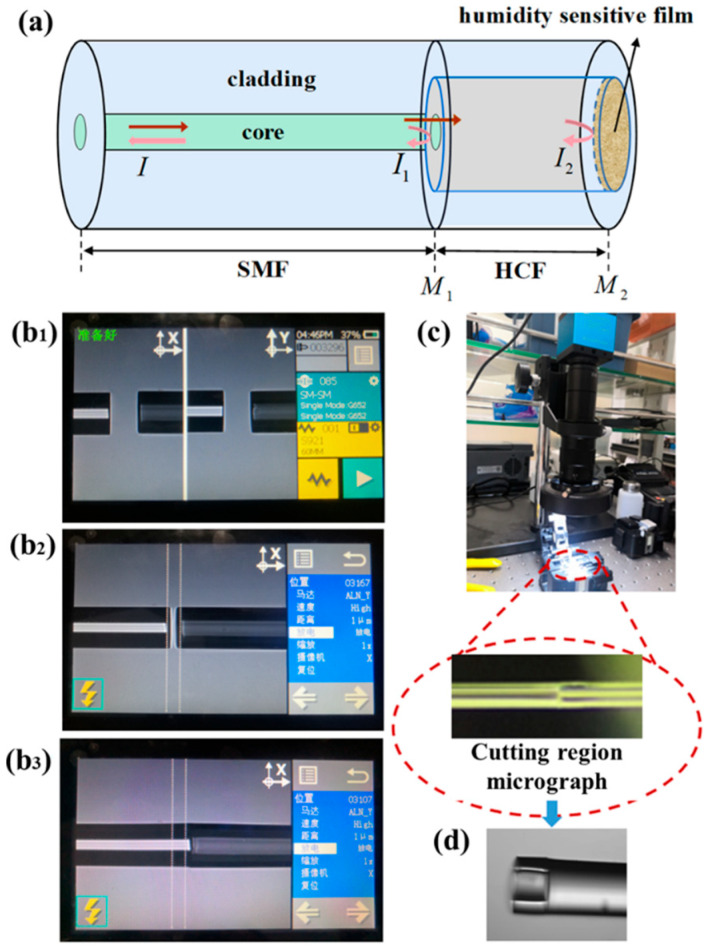
FPI humidity fiber sensor and fabrication process of SMF–HCF cascade-spliced structure. (**a**) Schematic diagram of FPI optical fiber humidity sensor structure based on humidity sensitive material; microscope (**b_1_**–**b_3_**) Diagrams of fusion splicing process; (**c**) Real-time cutting and monitoring system; (**d**) practical sensing probe.

The optical fiber sensing architecture, which has been schematically illustrated in Figure 1, incorporates an FPI microcavity formed by precision cleaving and splicing an HCF with an SMF, followed by coating a hygroscopic polymer film to encapsulate the FPI cavity opening. Variations in ambient relative humidity induce volumetric and refractive index changes in the hygroscopic film through water molecule interactions, thereby modulating the interference phase of reflected spectra. Real-time humidity monitoring will be achieved by analyzing the wavelength shifts in the FP interference spectra.

Within this FPI fiber microcavity humidity sensor, the two reflective interfaces defining the FPI cavity are the splicing interface of SMF–HCF and the hygroscopic polymer film. The spectrum quality of FPI cavity configuration relies on the precise HCF length control and its high-quality splicing with SMF, as well as the film thickness and uniformity.

### 2.1. Fiber Structure Fabrication

The fabrication process of SMF–HCF cascade-spliced structure is outlined in Figure 1b_1_–b_3_. Here, the coating layers of SMF and HCF were removed. The acrylate coating of the SMF (YOFC G652D: core diameter 8.2 μm, cladding diameter 125 μm) was mechanically stripped using fiber strippers, while for the HCF (Polymicro Technologies TSP series: inner diameter 50 μm, outer diameter 146 μm) coating layer, its polyimide coating layer was thermally removed via flame heating to preserve the integrity of the hollow-core structure. This procedure ensures the optimal light confinement and interfacial reflectivity for generating the high-fidelity FPI signals and demining the humidity sensing performance. This is because the optical signal emitted from the SMF core will diverge within the HCF, whose length and diameter should be strictly limited to avoid the optical signal entering its tube-wall and exciting higher-order modes. In this case, the Mach–Zehnder interference (MZI) or multi-mode interference (MMI) effect will be produced [26]. Therefore, the length and diameter of HCF were seriously controlled and selected. The interference spectra for the inserted HCFs with the lengths of ~70 μm, ~100 μm, ~150 μm, ~200 μm, and ~300 μm have been compared. The interference spectra of the first two lengths have a larger free spectral range (FSR) and fewer interference periods in the full spectral range. The extinction of the interference spectra of the latter two lengths is relatively small due to the leakage of optical signals into the sidewalls of HCF. In the aspect of the sensitivity of the proposed FPI humidity sensor, it is dependent on the type of humidity sensitive materials and the relative change in the length of the FP cavity (HCF length). Shorter length of HCF results in a higher sensitivity. To obtain a higher extinction and a proper interference period (or FSR), the HCF was cut with the desired length of ~140 μm ± 10 μm by precisely mutually moving the fiber holders under the real-time monitoring of a microscope (as Figure 1c shows), while the air core diameter of 50 μm and the outer diameter of 146 μm can effectively avoid the collapse-damage of hollow structure and reduce the optical losses near the SMF–HCF splicing interface.

The splicing operation was conducted using a FITEL S179 fusion splicer, wherein SMF and HCF with polished end-faces were secured in electrode-mounted clamps. Critical splicing parameters, including discharge intensity, duration, fiber advancement distance, and electrode offset, were systematically optimized. Due to the comparable mode field areas of HCF (50 μm inner diameter) and SMF, the single-discharge processing will result in satisfactory splicing quality. However, conventional automatic alignment protocols proved ineffective due to the HCF’s enlarged air core, which risked collapse under excessive current. Consequently, a manual splicing strategy was implemented as follows: (1) Horizontal alignment was achieved via Z_L/Z_R motor adjustments with 5 μm step resolution to position fibers near electrodes. (2) Vertical alignment was performed through ALN_X/ALN_Y motor movements at 1 μm precision to ensure coaxial end-face alignment. (3) Discharge parameters were empirically optimized through iterative experimentation and validation of the literature, with optimal splicing outcomes achieved at 10-bit discharge intensity (≈15.6 mA) and 200 ms duration. This configuration minimized the air core deformation while maintaining the structural integrity, as illustrated in Figure 1b_1_–b_3_. The SMF–HCF splicing interface subsequently functioned as the primary reflective surface of the FPI cavity. This methodology ensures the precision alignment and controlled energy delivery, effectively balancing the splicing reliability with preservation of the HCF’s hollow structure.

The HCF was positioned on a precision cleaver (Vytran LDC400, Thorlabs Inc., Newton, NJ, United States) for cutting with the desired length. Real-time microscopic imaging (Figure 1c) enabled the visual tracking of the splice junction when manually splicing the different fibers. The significant core diameter disparity between SMF (8.2 μm) and HCF (50 μm air core) facilitated that the splicing interface can be rapidly identified. Upon alignment of the junction with the cleaver blade axis, the fiber was immobilized and cleaved to achieve a predefined HCF length with an optically flat end-face, essential for subsequent coating processes. The prepared HCF tip was illustrated in Figure 1d, retained the SMF–HCF splice interface (M1), and exposed a pristine HCF surface for hygroscopic film deposition.

### 2.2. PMMA Film Coating

To develop the FPI humidity sensor, the polymer film will be elaborated onto the opening hole of the SMF–HCF tip by the dip-coating method. Firstly, the hygroscopic polymer solution will be prepared. In this section, taking PMMA film for example, the polymer solution preparation and coating process will be explained in detail. A measurement of 0.2 g/mL PMMA sol solution was prepared by dissolving 4 g PMMA pellets (Mw ≈ 120,000) in 20 mL acetone under 40 kHz ultrasonication for 5 h. The fiber assembly was vertically mounted on a dip-coating apparatus (SYDC-100, Shanghai Sanyan Technology Co., Ltd., Shanghai, China) with the HCF end suspended above the PMMA solution. The immersion and withdrawal at 50 μm/s enabled the uniform film formation on the HCF end-face, establishing the second reflective interface required for FPI cavity functionality.

**Figure 2:**
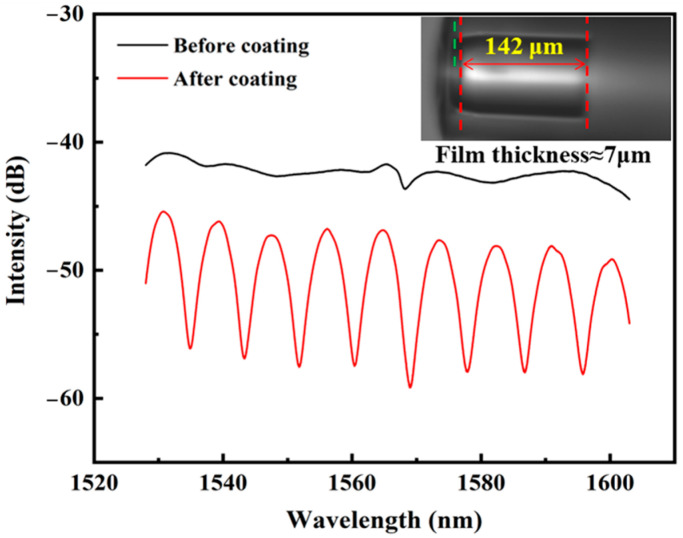
Spectrum comparison for optical fiber structure before and after coating.

In situ spectral monitoring via an optical spectrum analyzer (OSA, Yokogawa AQ6370D, Yokogawa Electric Corporation, Tokyo, Japan) can confirm the FP cavity formation. Bare HCF structures exhibited featureless reflection spectra, whereas post-coating measurements (Figure 2) revealed distinct interference fringe patterns with multiple resonant peaks and troughs, verifying the successful fabrication for the FPI probe. This real-time spectral analysis method ensured the optimal film thickness control (≈2.5 μm per coating cycle) while preventing over-deposition-induced optical loss.

The finalized dual-reflective FPI architecture leverages the light reflection at the SMF–HCF interface and PMMA–air boundary to generate the humidity sensitive interference spectra, with the refractive index and position modulated by PMMA–water molecule interactions. In this experiment, thinner polymer films are difficult to form the interference spectra with sufficient extinction ratio, while thicker films will increase optical losses. Moreover, in order to maintain the high humidity sensitivity, the film thickness should not be too large. The film thickness was precisely controlled during the dip-coating process through the coating cycles; each cycle will introduce a further thickness of ~2.5 μm, which can be confirmed by calculating the free spectrum range (FSR) of the interference spectrum. Here, the increasing HCF length (i.e., FP cavity length, *L*) progressively reduced the FSR, consistent with the theoretical relationship:

(1) $$FSR\;\;=\;\;\frac{\lambda^{2}}{2nL}$$

where *λ* refers to interference wavelength; *n* is the refractive index of the FP cavity (*n* = 1 corresponds to air); and *L* is FP cavity length, naming the distance between the SMF–HCF interface and polymer film surface.

## 3. Performance Characterization of PMMA-FPI Fiber Optic Humidity Sensor

**Figure 3:**
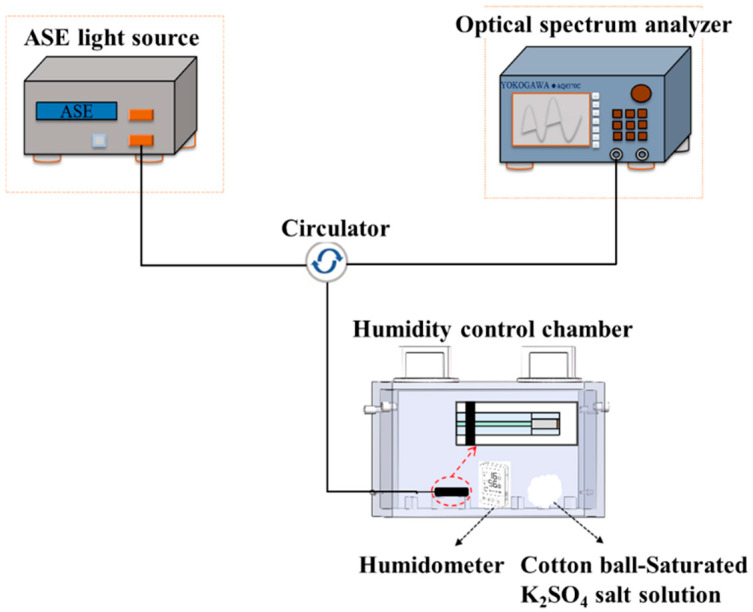
Schematic diagram of optical fiber humidity sensor test system.

The humidity sensing evaluation system, schematically depicted in Figure 3, comprised an ASE broadband light source (KG-ASE-CL-D-13-FC/APC, Kangguan Technology Co., Ltd., Shenzhen, China, 1528–1603 nm wavelength range), an OSA, and a custom 2 L hermetic chamber to place the FPI sensor probe, a digital hygrometer (0.1% RH resolution), and humidity-regulating saturated K_2_SO_4_ solution. Relative humidity (RH) modulation was achieved through controlled evaporation from K_2_SO_4_-impregnated cotton balls, with the hygrometer providing real-time RH calibration adjacent to the sensing probe.

The PMMA-coated FPI (PMMA-FPI) sensor was optically coupled via a three-port fiber circulator to enable reflection-mode interrogation. The light was launched from the ASE source and propagated through the circulator to the sensor, with reflected interference signals routed to the OSA for spectral analysis. Hygroscopic swelling of the PMMA film induced humidity-dependent refractive index and geometric changes, manifesting as spectral blue-shifts in the FP interference pattern. To mitigate thermal cross-sensitivity, the chamber temperature fluctuation was stabilized at ±0.5 °C using a thermoelectric controller. The spectral stability was confirmed under equilibrium conditions, followed by complete sealing of the chamber via threaded caps and valve closure. The spectra were acquired starting at the humidity of 35% RH, with OSA recordings performed at 5% RH in increments up to 65% RH. During this humidity range, the K_2_SO_4_-impregnated cotton balls can supply the stable changing humidity environment.

**Figure 4:**
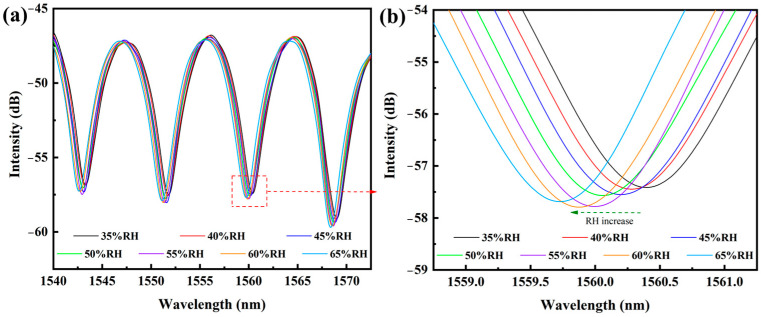
Spectrum response of PMMA-FPI humidity sensor during humidity range of 35–65% RH; (**a**) whole interference spectrum, and (**b**) local magnification near 1560 nm.

Post-processing of spectral data involved the Savitzky–Golay smoothing algorithm and recording the wavelength of a special resonance dip. The Savitzky–Golay smoothing algorithm was adopted in the Origin 2024 software with the window size of 5 points and the second-order polynomial fitting to eliminate the random noise signals. Figure 4a illustrates the characteristic interference spectra across the 35–65% RH range, exhibiting progressive wavelength shifts and fringe visibility changes correlating with PMMA film hydration states. This systematic characterization methodology ensures the quantitative evaluation of humidity sensitivity while minimizing environmental interference factors. It should be noted that the broadband light source and frequency-domain interference spectrum used in this experiment can facilitate the evaluation of static parameter information, such as sensitivity and interference spectrum quality of the sensing probe. In the practical application of the fiber optic humidity sensor, a narrowband laser with a specific wavelength will be used as the light source and the interfering intensity at this fixed wavelength will be real-time traced by the fast photodetector to calculate the humidity fluctuation.

The wavelength location for one special interfering peak was recorded in the optical spectrum analyzer or the Origin software to establish the relationship between the wavelength and the humidity change. As illustrated in Figure 4a, the interference spectra exhibited the conserved fringe morphology with systematic wavelength shifts under increasing humidity. Magnified analysis of the 1560 nm trough (Figure 4b) revealed a linear blue-shift from 1560.416 nm to 1559.72 nm across the 35–65% RH range, corresponding to a humidity sensitivity of 0.023 nm/%RH. This behavior originates from the hygroscopic expansion of PMMA film, which reduces the FPI cavity length (Δ*L* ≈ *λ*Δ*n*/2*n*, where *n* denotes effective refractive index) through combined thickness and refractive index modulation. The observed directional spectral shift aligns with the theoretical predictions for hydrophilic polymer-coated FPI sensors.

## 4. Comparative Analysis of Hygroscopic Material Performance

To evaluate the material-dependent sensing properties, two additional hydrophilic films were investigated, respectively, for PVA with 8 wt% aqueous solution (4.35 g PVA powder in 50 mL deionized water) and PEG with 9% (*w*/*v*) solution (2 g PEG in 20 mL deionized water). Both aqueous formulations exhibited higher mass concentrations than the PMMA-acetone system (0.2 g/mL) while maintaining lower viscosity.

**Figure 5:**
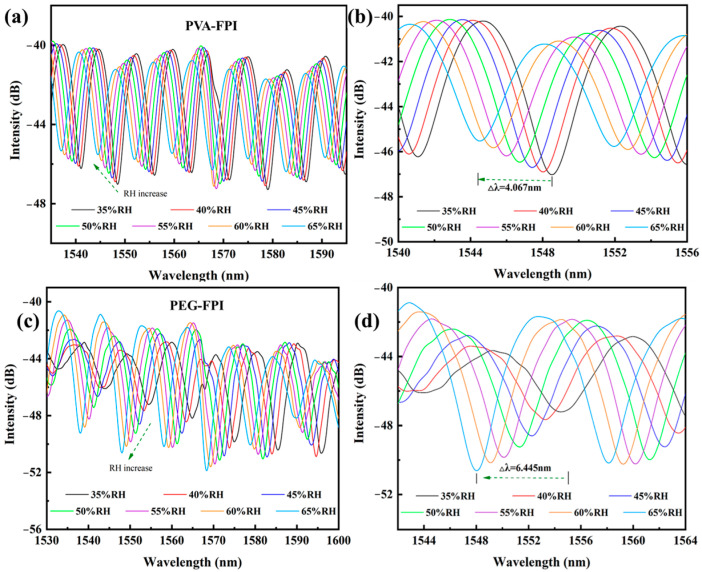
Humidity sensing characteristics of PVA-FPI (**a**,**b**) and PEG-FPI (**c**,**d**) humidity sensors.

The identical humidity testing apparatus was employed for PVA-FPI and PEG-FPI probes. Figure 5a,b presents the characteristic interference spectra of PVA-FPI and PEG-FPI sensors, respectively, demonstrating distinct humidity-induced spectral modulation patterns compared to those of PMMA-FPI. The experimental results reveal the spectral shift linearity of PMMA > PVA > PEG. This systematic comparison highlights PMMA’s superior stability and measurement repeatability despite slower response kinetics, attributed to its cross-linked polymer matrix and reduced water solubility relative to PVA/PEG systems. The aqueous-based sensors, while faster-responding, suffered from partial film dissolution during prolonged high-humidity exposure (>75% RH), necessitating surface modification for practical deployment.

Humidity measurement experiments demonstrated the systematic blue-shifts in sensor reflection spectra across the 35–65% RH range. While intensity variations observed were attributable to humidity-modulated refractive index changes in hygroscopic PVA films, spectral modulation predominantly manifested as wavelength displacement. Figure 5b details localized spectral analysis near the 1548 nm interference trough, revealing a total wavelength shift of 4.067 nm with negligible intensity modulation (<2%), confirming the appropriateness of wavelength demodulation for sensitivity quantification. PEG-FPI sensor characterization (Figure 5c,d) exhibited an analogous blue-shift behavior under increasing humidity, accompanied by non-systematic intensity fluctuations (≈15%). These intensity variations stem from the complex phase modulation mechanisms. Here, hydration-induced refractive index changes (Δn ≈ 0.012 per 10% RH) in PEG films alter interference conditions between reflected beams, modifying fringe visibility through the relationship:

(2) $$V\;\;=\;\;\frac{2\sqrt{I_{1}I_{2}}}{I_{1}\;\;+\;\;I_{2}}\cos\left(\Delta \phi \right)$$

where Δ*ϕ* = 4π*nL*/*λ* represents the phase difference.

**Figure 6:**
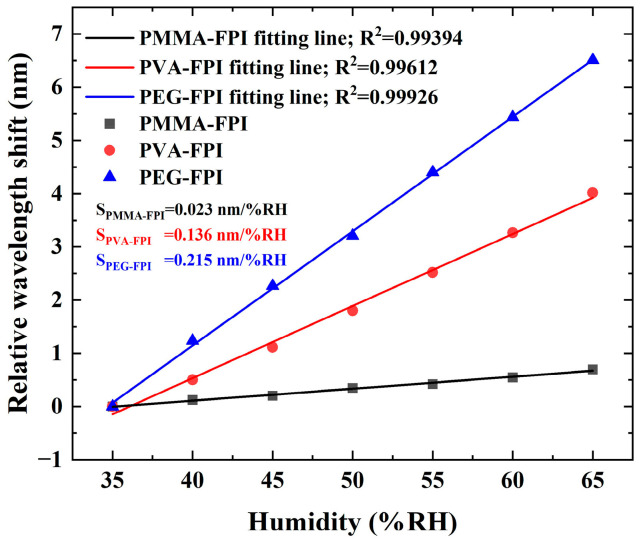
Depending relationship between the relative humidity and interference dip wavelength for FPI optical fiber sensors based on three different humidity sensitive materials.

To quantitatively compare humidity responsiveness, the linear regression analysis was applied to trough wavelength displacements from localized spectra (Figure 4b and Figure 5b,d). Figure 6 presents the normalized sensitivity curves for all three type sensors, where the sensitivities are 0.023 nm/%RH (*R*^2^ = 0.99394) for PMMA-FPI, 0.136 nm/%RH (R^2^ = 0.99612) for PVA-FPI, and 0.215 nm/%RH (*R*^2^ = 0.99926) for PEG-FPI. Depending on the resolution (20 pm) of OSA used in this experiment, the corresponding resolutions for three polymer materials are 0.870% RH, 0.147% RH, and 0.093% RH, respectively.

This hierarchy reflects different hydration dynamics for the specific materials. PMMA with the cross-linked structure restricts swelling (Δ*L*/*L* ≈ 0.4%/RH); PVA with the hydroxyl-rich matrix enables moderate expansion (Δ*L*/*L* ≈ 2.36%/RH); PEG has the linear polymer chains and permits the significant water absorption (Δ*L*/*L* ≈ 3.74%/RH). The inverse relationship between sensitivity and measurement stability highlights the critical material selection trade-offs for practical humidity sensing applications.

Notably, PEG-FPI exhibited a total wavelength shift of 6.445 nm (35–65% RH) compared to minimal intensity variation (3.528 dB), reaffirming wavelength demodulation as the optimal signal extraction method. The observed sensitivity hierarchy (PEG > PVA > PMMA) correlates directly with material hydrophilicity and polymer chain mobility, where

(3) $$S_{\lambda }\;\;\propto \;\;\frac{\partial n}{\partial RH}\;+\;\frac{n}{L}\frac{\partial L}{\partial RH}$$

with *S_λ_* denoting wavelength sensitivity.

## 5. Discussions

**Figure 7:**
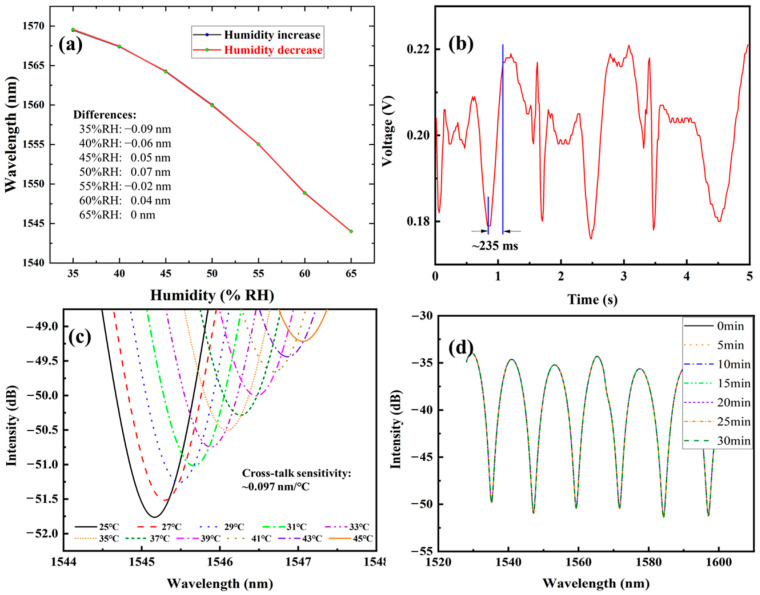
Sensing performance of SMF–HCF–PEG-based FPI humidity sensor. (**a**) Hysteresis; (**b**) Dynamic response; (**c**) Temperature cross-talk; (**d**) Stability.

In addition to humidity sensitivity, other performance parameters can also determine the actual application for the proposed humidity sensor. In this section, the performance in hysteresis, dynamic response, temperature cross-talk sensitivity and long-term stability of the PEG-based humidity sensor have been studied, as illustrated in Figure 7.

The humidity was increased from 35% RH to 65% RH, then decreased back to 35% RH; meanwhile, the wavelength location of one special interference was recorded. As Figure 7a shows, the hysteresis error ranges from 0.04 to 0.09 nm, exerting an uncertainty of 0.186–0.419% RH for the humidity measurement. To value the dynamic response, a 1550 nm laser was launched into the fiber sensor and an oscilloscope was used to record the real-time changes in light intensity. The humidity change was provided by the respiratory with the frequency of ~2.5 Hz. Figure 7b revealed the response time of ~235 ms. Environmental temperature will exert an impact on the humidity sensing performance. When the temperature changed from 25 °C to 45 °C, the wavelength underwent red-shift with a cross-talk sensitivity of ~0.097 nm/°C in Figure 7c. Compared to the humidity sensitivity, the introduced error is ~0.451% RH/°C. The temperature cross-sensitivity of the fiber optic humidity sensing system can be effectively compensated by introducing a temperature sensor into the fiber optic system to obtain the change in temperature, such as FBG, which is only sensitive to the ambient temperature. Then, based on the known temperature changes and temperature cross-sensitivity coefficient, the calibration quantity can be calculated to realize the temperature compensate and reduce its impact on optical interfering phase. Figure 7d shows the stability of the humidity sensor through continuous observation of the wavelength location for 30 min at the fixed temperature and humidity condition. The wavelength is relatively stable within 30 min, with a maximum fluctuation range of ~20 pm. According to the humidity sensitivity of 0.215 nm/% RH, this fluctuation corresponds to a relative humidity fluctuation of 0.093% RH, which can be ignored in most practical applications.

The sensing performance of the proposed humidity sensor has been compared with that of other reported works in Table 1.

As discussed in the Section 1, the typical humidity fiber sensors are developed by elaborating the humidity sensitive materials either on the outer surface of the cascade-spliced fiber structures based on MMF, HCF, photonic crystal fiber, no-core fiber, etc., or on the tip-end of the fiber structures. For the first case, optical fiber can be side-polished as a D-shaped structure [20] or bent to be a droplet structure [21] to launch out the light signal from the fiber core and produce the MZI, MMI, or evanescent wave effect [22] to improve the interface efficiency between the light and the outer environment. Furthermore, the metal nanostructure can be introduced to excite the surface plasmon effect to improve the humidity sensitivity [20]. Compared with silica capillary [23], the HCF with a similar diameter to SMF can contribute more stable sensing performance.

## 6. Conclusions

This study presents a family of hygroscopic polymer-coated FPI fiber optic humidity sensors fabricated through dip-coating methodology. The HCF cavity can be precisely controlled (140 ± 2 μm) via mechanical fusion splicing. The humidity sensitivity during 0.023–0.215 nm/% RH and linearity of R^2^ > 0.99 have been experimentally demonstrated for PMMA, PVA, and PEG polymer films. All sensors demonstrated consistent blue wavelength shifts under humidity elevation. PEG-FPI achieves the maximum sensitivity through its linear polymer architecture facilitating rapid water absorption. Comparative analysis revealed the fundamental trade-offs between sensitivity and measurement stability. The proposed sensor platform offers the significant practical advantages of simplified fabrication via dip-coating, compact footprint compatible with confined spaces, cost-effective production, and customizable response through material selection. These findings establish polymer-coated FPI sensors as viable solutions for precise humidity monitoring in industrial process control, agricultural storage, and biomedical applications. Future work will focus on multiplexed sensor arrays and surface functionalization for enhanced selectivity in multi-parameter environments.

## Figures and Tables

**Table 1:** Sensing performance comparison of polymer-based fiber humidity sensors.

Structure	Sensitivity	Resolution	Range	Response	Ref.
D-shaped fiber Au/PVA film	4.98 nm/% RH	/	40–90	440 ms	[20]
Droplet-like SMF PVA film	−0.6 nm/% RH	0.971% RH	35–95% RH	1.33 ms	[21]
MMF PMMA film	188.3 lux/% RH	/	5–95% RH	8 s	[22]
SMF-capillary cellulose film	1.24 nm/% RH	/	48–60% RH	1.61 s	[23]
SMF–HCF PEG film	0.215 nm/% RH	0.093% RH	35–65% RH	~235 ms	This work

## Data Availability

The original contributions presented in this study are included in the article. Further inquiries can be directed to the corresponding author.

## References

[1] Elsherif M., Salih A.E., Muñoz M.G., Alam F., AlQattan B., Antonysamy D.S., Zaki M.F., Yetisen A.K., Park S., Wilkinson T.D. (2022). Optical fiber sensors: Working principle, applications, and limitations. Adv. Photonics Res..

[2] Jean-Ruel H., Albert J. (2024). Recent advances and current trends in optical fiber biosensors based on tilted fiber Bragg gratings. TrAC Trends Anal. Chem..

[3] Ngiejungbwen L.A., Hamdaoui H., Chen M.Y. (2024). Polymer optical fiber and fiber Bragg grating sensors for biomedical engineering Applications: A comprehensive review. Opt. Laser Technol..

[4] Gan J., Yang A., Guo Q., Yang Z. (2024). Flexible optical fiber sensing: Materials, methodologies, and applications. Adv. Devices Instrum..

[5] Jha R., Gorai P., Shrivastav A., Pathak A. (2024). Label-free biochemical sensing using processed optical fiber interferometry: A review. ACS Omega.

[6] Kumar V., Raghuwanshi S.K., Kumar S. (2022). Advances in nanocomposite thin-film-based optical fiber sensors for environmental health monitoring—A review. IEEE Sens. J..

[7] Upadhyay S., Kumar A., Srivastava M., Srivastava A., Dwivedi A., Singh R.K., Srivastava S.K. (2024). Recent advancements of smartphone-based sensing technology for diagnosis, food safety analysis, and environmental monitoring. Talanta.

[8] Cheng T., Li B., Zhang F., Chen J., Zhang Q., Yan X., Zhang X., Suzuki T., Ohishi Y., Wang F. (2022). A surface plasmon resonance optical fiber sensor for simultaneous measurement of relative humidity and temperature. IEEE Sens. J..

[9] Liu L.L., Morgan S.P., Correia R., Korposh S. (2022). A single-film fiber optical sensor for simultaneous measurement of carbon dioxide and relative humidity. Opt. Laser Technol..

[10] Li J., Yan H., Dang H.T., Meng F.L. (2021). Structure design and application of hollow core microstructured optical fiber gas sensor: A review. Opt. Laser Technol..

[11] Liu H.H., Hu D.J.J., Sun Q.Z., Wei L., Li K.W., Liao C.R., Li B.Z., Zhao C., Dong X.Y., Tang Y.H. (2023). Specialty optical fibers for advanced sensing applications. Opto-Electron. Sci..

[12] Liu W., Liu Z.H., Zhang Y., Li S., Zhang Y.X., Yang X.H., Zhang J.Z., Yuan L.B. (2022). Specialty optical fibers and 2D materials for sensitivity enhancement of fiber optic SPR sensors: A review. Opt. Laser Technol..

[13] Chen M.Q., Zhao Y., Wei H.M., Zhu C.L., Krishnaswamy S. (2021). 3D printed castle style Fabry-Perot microcavity on optical fiber tip as a highly sensitive humidity sensor. Sens. Actuators B Chem..

[14] Li S., Wan T., Wei H.G., Wang S.Y., Wang B., Cheng B.W. (2022). Flexible highly-sensitive humidity sensor based on CGO/SMPLAF for wearable human skin humidity detection. Sens. Actuators B Chem..

[15] Dias B., Carvalho J., Mendes J.P., Almeida J.M., Coelho L.C. (2022). Analysis of the relative humidity response of hydrophilic polymers for optical fiber sensing. Polymers.

[16] Zhang Y., Wu Y., Fu Y., Jia Q.X., Zhang Z. (2024). Sulfonated hypercross-linked porous organic polymer based humidity sensor. Sens. Actuators B Chem..

[17] Montes-García V., Samorì P. (2024). Humidity sensing with supramolecular nanostructures. Adv. Mater..

[18] Syuhada A., Shamsudin M.S., Daud S., Krishnan G., Harun S.W., Aziz M.S.A. (2021). Single-mode modified tapered fiber structure functionalized with GO-PVA composite layer for relative humidity sensing. Photonic Sens..

[19] Guo Y., Wei J.Q., Yu Z.W., Wang J.Y., Chen X.D., Wang J. (2024). Moisture modulated refractive index difference of engineered pore interface for indoor temperature and humidity regulation. Chem. Eng. J..

[20] Wang Y., Wang J.R., Shao Y., Liao C.R., Wang Y.P. (2021). Highly sensitive surface plasmon resonance humidity sensor based on a polyvinyl-alcohol-coated polymer optical fiber. Biosensors.

[21] Al-Hayali S.K., Salman A.M., Al-Janabi A.H. (2021). Effect of hygroscopic polymer-coatings on the performance of relative humidity sensor based on macro-bend single-mode fiber. Opt. Fiber Technol..

[22] Li J.Z., Lai M., Zhang H.H., Song H.T., He J.X., Chen Y.X., Qi Y.T., Zhu B., Ma Y., Liu B. (2023). Performance-enhanced fiber optic humidity sensors based on SiO_2_/porous PMMA coatings. Appl. Opt..

[23] Yan H., Sun D.D., Hou Z.F., Wang G.J. (2023). Miniature and high-performance optical fiber relative humidity sensor based on Fabry-Perot structure interacting with hydroxyethyl cellulose. Opt. Mater..

[24] Wang Y.N., Li J., Zhang Q., Gao X.H., Yuan Z.Y., Meng F.L. (2025). Ultraviolet-sensitized room temperature fiber optic evanescent field ammonia sensor with polyacrylamide/metal organic framework-carbon quantum dots (–COOH) gas-sensitive film. J. Colloid Interface Sci..

[25] Wang Y.N., Li J., Yuan Z.Y., Meng F.L. (2025). Optical fiber triethylamine room temperature gas sensor based on HMS platform with DFT calculation and experimental demonstration. Appl. Surf. Sci..

[26] Liu R.J., Zhao Y., Zhou Z., Gao R., Lv R.Q., Ma Y.X. (2026). Lab-on-fiber: A novel high-sensitivity Mach-Zehnder interferometer with dual-sensing cavity in hollow-core fiber for simultaneous measurement of seawater temperature and salinity. Sens. Actuators B Chem..

